# Factor structure and psychometric validation of the Work and Well-being Inventory

**DOI:** 10.3389/fpubh.2026.1816119

**Published:** 2026-06-24

**Authors:** Christine E. Garver-Apgar, Zachary Giano, Ashley M. Kayser, Chad D. Morris

**Affiliations:** 1 Department of Psychiatry, School of Medicine, University of Colorado Anschutz Medical Campus, Aurora, CO, United States; 2 Department of Biostatistics and Informatics, Colorado School of Public Health, University of Colorado Anschutz Medical Campus, Aurora, CO, United States

**Keywords:** assessment, employee well-being, factor structure, inventory, occupational health, psychometric validation, wellness, workplace culture

## Abstract

**Background:**

This study examined psychometric properties of a novel measure of occupational health and wellness – the Work and Well-being Inventory (WWI). Unlike other validated measures of employee well-being, the WWI adopts a holistic, eight-dimension model of wellness, delineates personal wellness from employer-supported wellness, and is straightforward to interpret.

**Methods:**

Participants (*n* = 949) were a convenience sample of employees from four large behavioral health organizations across Illinois, Missouri, Nebraska, and Texas. As part of a broader wellness initiative, employees at all levels across each organization were invited and encouraged by organizational leadership to complete a web-based, self-report survey, administered via Qualtrics, over a 2-week period in spring of 2022 or summer of 2023. Employee participation rates ranged from 41 to 81%, depending on organization. Analyses focused on evaluating factor structure of the WWI with confirmatory factor analysis using separate developmental and validation groups. We also assessed convergent and discriminant validity by correlating subscales with established measures of related constructs.

**Results:**

The final inventory contains 106 items comprising 16 subscales which can be administered independently of each other (eight “personal” wellness dimensions and eight “employer-supported” wellness dimensions). This model demonstrated good fit to the data and internal consistency. Patterns of correlations between subscales and external measures were consistent with theoretical expectations, supporting both convergent and divergent validity of constructs.

**Conclusion:**

The WWI is a validated measure of employee well-being that adopts an “eight dimension” model of wellness. Factor structure and model fit statistics support flexible administration and parsimonious interpretation of eight subscales directly corresponding to eight dimensions of wellness. The instrument has utility for researchers investigating occupational health promotion and organizations seeking to evaluate initiatives designed to improve employee wellness, retention, and workplace culture.

## 1 Introduction

The concept of wellness extends beyond the absence of disease to encompass a holistic combination of domains including physical and emotional health, social wellness, and others (1). Interest in the wellness of workers is increasing across organizations, consultancy firms, and academia. Healthcare and public health workers have, not surprisingly, been a focus for wellness initiatives over the past five years, given pandemic-related stress and deterioration of working conditions (2). Efforts to measure impacts of employer-led well-being interventions are becoming increasingly frequent across all sectors, resulting in a proliferation of occupational wellness assessments with varying levels of methodological quality. A recent systematic review determined that none of the 18 well-being instruments developed between 2010 and 2020 and selected for the review met rigorous criteria of the international Consensus-based Standards for the selection of health Measurement Instruments (3).

Comprehensive models for understanding and informally assessing individuals’ wellness conceptualize wellness along seven or eight dimensions (or pillars): emotional, social, physical, intellectual, occupational, spiritual, environmental, and for most, financial. These models emerged from a convergence of theories in public mental health, positive psychology, and systems science (1, 4–10), and are utilized across a range of sectors including behavioral health (e.g., Substance Abuse and Mental Health Administration), higher education (e.g., Princeton University, University of Colorado Boulder), and law (e.g., New York State Bar Association, Wisconsin State Bar Association). Although broadly recommended for self-reflection, assessments using an eight dimensions framework have received little psychometric attention, limiting their use for research, clinical, or quality improvement purposes. An exception is the 67-item Wellness Inventory, which was recently demonstrated to have good internal consistency, test–retest reliability, and reasonable construct (11).

Existing assessments based on the eight dimensions of wellness are valuable for measuring holistic, individual wellness; however, they do not capture the extent to which individuals’ wellness is supported by their employers or places of work. Many validated assessments exist to measure workplace wellness, conditions at work, supportive organizational practices, or other organization/employer-focused factors to improve employee wellness or retention (12–17). However, very few of these scales measure wellness from multiple dimensions similar to those described above. Moreover, workplace well-being scales often do not provide practical, actionable feedback for organizations that wish to implement employee wellness initiatives or improve their culture. Because items often assess general constructs [e.g. “I am emotionally energized at work” (16) or “My organization is committed to employee health and well-being” (17)], negative scores do not necessarily provide clear direction for change to organizational leadership and/or wellness champions.

The National Institute for Occupational Safety and Health Worker Well-Being Questionnaire (NIOSH WellBQ) is an example of one assessment which does measure worker wellness from multiple dimensions and has undergone rigorous psychometric validation (17). However, some features may limit the NIOSH WellBQ’s utility in certain contexts. It contains very few items assessing wellness outside of the work setting, which limits important contextual information for organizations and researchers. Moreover, not all five domains include items that would suggest specific action steps organizations could take in response to negative scores. As well, the majority of the NIOSH WellBQ uses 4-point response scales, which prevents the measurement of true neutrality and could lower response variation and reduce measurement precision. Importantly, the NIOSH WellBQ is highly complex (containing 5 domains, 24 sub-domains, and 52 sub-domain constructs), rendering analysis and interpretability difficult and time-consuming across many contexts.

It is important to distinguish the Work and Well-being Inventory (WWI) from the similarly named Work and Well-Being Inventory (WBI) developed by Vendrig and Schaafsma (18). Although both instruments assess aspects of work and well-being, their underlying purposes and conceptual frameworks differ substantially. The WBI was developed as a screening tool in occupational health and rehabilitation contexts, primarily aimed at identifying psychosocial risk factors associated with sickness absence and return-to-work outcomes, with domains focused on stressors, symptoms, coping, and illness behavior. In contrast, the WWI is grounded in a holistic, multidimensional model of wellness and is designed to assess both personal and employer-supported wellness across eight domains, with an emphasis on interpretability and actionable feedback for organizations. As such, the WWI extends beyond risk identification to provide a comprehensive framework for understanding and improving employee well-being.

The current study was designed to evaluate the Work and Well-being Inventory (WWI), a measure created and tested for healthcare and public health organizations seeking to improve employee wellness and retention using actionable data. Initially developed in 2011, the WWI brings together several theories and models across psychology and public mental health: holistic health (4), Hettler’s multidimensional model (5), Engel’s biopsychosocial paradigm (6), positive psychology and recovery-oriented behavioral health approaches (1, 7, 8), and organizational systems change (9, 10). Collectively, these theories view wellness as a dynamic, multidomain process that moves beyond only physical wellness.

Each dimension (physical, emotional, social, spiritual, etc.) represents a necessary but insufficient component of overall wellness; imbalance in one affects the others. Importantly, change happens both at the individual level and as a property of the system. Sustainable improvement requires redesigning work, leadership, incentives, and culture; and not asking workers to cope with broken systems. The WWI differs from existing workplace wellness measures (e.g., the Occupational Health and Well-being Questionnaire, TOMH well-being scale, and related instruments assessing workplace conditions or general well-being; refs 12–18) in several ways: 1. It adopts an eight-dimension model of wellness, measuring each dimension as a separate subscale for flexibility of administration and ease of interpretability; 2. It frames employee wellness as something both individuals *and* employers/organizations have agency over and assumes that individuals could score differently when considering their overall personal wellness versus their employer-supported wellness; thus the scale provides separate assessments for personal and employer-supported wellness with equal attention paid to each; and 3. Within every dimension, employer-supported wellness items include several *specific* factors so that data provide healthcare and public health leaders with concrete direction for improvements (e.g., “I receive recognition for a job well done”).

The WWI takes less than 10 min to complete, and it has been broadly utilized for health systems change projects across behavioral and public health organizations, supporting the tool’s face validity and establishing it as a practically useful assessment to drive quality improvement initiatives and change workplace culture. However, no studies have evaluated the WWI’s psychometric properties. The current study aimed to psychometrically evaluate the WWI using data collected from employees of four large behavioral health organizations in different states across the US, including model fit, internal consistency, and construct validity. Our goal was to empirically validate the WWI as a practical tool for use in healthcare, public health, and research settings.

## 2 Materials and methods

### 2.1 Sample

Participants (*n* = 949) were a convenience sample of employees at four large behavioral health organizations operating throughout Illinois, Missouri, Nebraska, and Texas. No incentives were provided for survey completion. All employees were encouraged to participate, regardless of role or seniority. The percentage of employees who chose to complete the survey ranged from 41 to 81%, depending on the organization. Combined demographics of survey respondents are provided in Table 1.

**Table 1:** Demographic characteristics.

Variable name	Full sample (*n* = 949)		Developmental group (*n* = 474)		Validation group (*n* = 475)		Sig.
	n/*μ*	%/SD	n/μ	%/SD	n/μ	%/SD	
Age (categorical)							0.059^a^
18–25	80	8.6	46	9.9	34	7.3	
26–35	225	24.1	100	21.5	125	26.7	
36–45	212	22.7	113	24.2	99	21.2	
46–55	219	23.4	119	25.5	100	21.4	
56–65	153	16.4	71	15.2	82	17.5	
66+	45	4.8	17	3.6	28	6.0	
Years with org	3.03	2.0	3.1	1.9	2.95	2.0	0.237^b^
Female	741	80.3	367	79.4	374	81.1	0.415^a^
Race/Ethnicity							0.071^a^
Asian	9	1	4	0.8	5	1.1	
Black/AA	65	6.9	41	8.7	24	5.1	
Hispanic/Latino	82	8.7	33	7.0	49	10.3	
NA/AK native	7	0.7	5	1.1	2	0.4	
White non-Hispanic	726	76.7	361	76.3	365	77.0	
Another race	22	2.3	14	3.0	8	1.7	
Prefer not to answer	36	3.8	15	3.2	21	4.4	
Organization role							0.923^a^
Full time salary	498	52.8	248	52.5	250	53.1	
Full time hourly	389	41.3	196	41.5	193	41.0	
Part time salary	6	0.6	2	0.4	4	0.8	
Part time hourly	32	3.4	15	3.2	17	3.6	
Manager/supervisor	7	0.7	4	0.8	3	0.6	
Sr. leadership	9	1	6	1.3	3	0.6	
Other	2	0.2	1	0.2	1	0.2	
Organization							0.083^a^
Organization #1	157	16.5	89	18.8	68	14.3	
Organization #2	367	38.7	190	40.1	177	37.3	
Organization #3	199	21.0	95	20.0	104	21.9	
Organization #4	226	23.8	100	21.1	126	26.5	
Job supports behavior change	641	67.7	329	69.6	312	65.8	0.219^a^

a Chi-Square.

b t-test.

### 2.2 Procedure

Data were collected as part of a larger initiative funded by Community Mental Health Centers to identify and improve employee wellness needs. Employees at all levels across each organization were invited and encouraged by organizational leadership to complete a web-based, self-report *Employee Wellness Survey*, administered via Qualtrics, over a 2-week period in spring of 2022 or summer of 2023. In most cases, employees were allotted time during work hours to complete the survey battery, which took approximately 15–20 min. This study was approved as a Quality Improvement (QI) Initiative by the Colorado Multiple Institutional Review Board and later amended to include a psychometric analysis of well-being data collected as part of the Employee Wellness Survey (COMIRB #22–0522). As a non-human subjects QI project, consent was waived.

### 2.3 Measures

#### 2.3.1 Work and Well-being Inventory (WWI)

The WWI is a multi-dimensional measure with 109 items that yields a quantitative score for each of eight dimensions, separately for personal and employer-supported domains, yielding a total of 16 subscales scores. Each item is positively framed, and respondents select their agreement with the statement on a 5-point Likert scale (“Strongly Disagree” to “Strongly Agree”). The scale is designed so that subscales can be administered as stand-alone assessments, depending on organizational need and interest. By separating personal wellness with employer-supported wellness, organizations and/or researchers can better contextualize impacts of workplace conditions on employee wellness.

To evaluate convergent and discriminant validity of the WWI, we collected data from participants using several additional measures. Unless otherwise cited, the following items were sourced from the Workplace Question Bank (19). Composite measures grouped items with conceptually similar constructs.

#### 2.3.2 World Health Organization-Five Well-being Index (WHO-5)

The WHO-5 (20) is a self-report instrument measuring mental well-being. It consists of five statements relating to the past two weeks. Each statement is rated on a 6-point scale; raw scores are multiplied by 4, such that total scores may range from 0 to 100, with higher scores indicating better mental well-being. A percentage score below 50 has been suggested as a cutoff for poor mental well-being and as an indication for further assessment for the possible presence of a mental health condition (21).

#### 2.3.3 Workplace PERMA Profiler

This measure is an adaptation of Seligman’s PERMA model and includes five items that capture the five pillars of a ‘flourishing’ life in a work setting: Positive emotion, Engagement, Relationships, Meaning, and Accomplishment (22, 23). Each statement is answered on a 11-point scale from ‘never’ or ‘not at all’ [0] through to ‘all of the time’ or ‘completely’ [10]. Composite scores are the average of the total for the five answers for each respondent. A value of 0 represents the least flourishing work life; a value of 10 represents the most highly flourishing state.

#### 2.3.4 Workplace demands (7-item subscale of Workplace Well-being Questionnaire)

This subscale (24) can be conceptualized as measuring the extent of intrusion of work into an employee’s private life. Example statements included, “My work eats into my private life” and “I feel stressed in organizing my work time to meet demands.” Participants responded to each item on a 5-point scale (“not at all true” to “extremely true”) and responses were summed across items, such that higher scores indicate higher workplace demands.

#### 2.3.5 Control/autonomy

This is a 4-item composite measure designed to assess the degree to which employees feel they have agency over how they conduct their professional activities: “I know what is expected of me at work,” “I am able to apply my own ideas in my work,” “I am consulted before objectives are set for my work,” and “I can influence decisions that are important for my work.” Participants responded to each item on a 5-point scale (‘never’ to ‘always’) and responses were summed across items such that higher scores indicate greater control/autonomy.

#### 2.3.6 Connection

This 3-item composite adapted from the UCLA Loneliness Scale (25) measures the extent to which employees feel a sense of connection vs. isolation at work: “I feel connected to my co-workers,” “I feel isolated in my job” (reverse-coded), and “I feel left out from things that are happening at my organization” (reverse-coded). Responses across a 5-point scale are summed, with higher scores indicating greater connection at work.

#### 2.3.7 Burnout

A single item, “Based on your definition of burnout, how would you rate your level of burnout?” Respondents select one of 5 options ranging from “I enjoy my work. I have no symptoms of burnout” to “I feel completely burned out and often wonder if I can go on. I am at the point where I may need some changes or may need to seek some sort of help” (26).

#### 2.3.8 Job satisfaction

A single item, “How dissatisfied or satisfied are you with your present job overall?” Responses are on a 7-point scale ranging from “completely dissatisfied” to “completely satisfied” with higher scores indicating greater job satisfaction.

#### 2.3.9 Recommend Job

A single item, “I recommend my organization as a great place to work.” Responses are on a 5-point scale ranging from “Strongly Disagree” to “Strongly Agree” with higher scores indicating higher job endorsement.

#### 2.3.10 Stress

A single item assessing perceived stress defines stress: “Stress means a state in which a person feels tense, restless, nervous, or anxious or is unable to sleep at night because his/her/their mind is troubled all the time. Do you feel this kind of stress these days?” Responses range across a 5-point scale from “not at all” to “very much”, with higher scores indicating greater perceived stress (27).

#### 2.3.11 General health

A single item, “How is your health in general?” Responses range from “very bad” to “very good” on a 5-point scale with higher scores indicating greater health.

### 2.4 Analytic strategy

To support a rigorous evaluation of the scale structure and ensure independent validation, participants were randomly assigned to either a Developmental Group (*n* = 474) or a Validation Group (*n* = 475). Randomization was carried out using a random number formula in Microsoft Excel, with approximately half of the sample assigned to each group. This approach aligns with recommendations by Worthington and Whittaker (28), who advocate for using separate samples for model refinement and cross-validation in scale development research. As shown in Table 1, the two groups did not differ significantly on key demographic characteristics, confirming successful randomization.

The study aimed to develop and validate a set of 16 self-report subscales assessing two domains of well-being: employer-supported and personal, with eight distinct constructs in each domain. Because the WWI was developed using a theory-driven, multidimensional framework in which each wellness dimension was designed to function as an independent subscale, we evaluated factor structure using confirmatory factor analysis (CFA) for each subscale separately rather than exploratory factor analysis of the full item pool. To guide the validation process, we followed the strong program of construct validation described by Benson (29) and operationalized by Bashkov and Coggeshall (30), which emphasizes three stages: substantive, structural, and external. During the substantive stage, items were developed based on theory and reviewed to ensure conceptual alignment with their respective constructs.

In the structural stage, we evaluated the psychometric properties of each scale through a series of CFAs conducted in the Developmental Group. Analyses were conducted in Mplus version 1.8.6 using maximum likelihood (ML) estimation. We applied the following criteria to determine model adequacy: (a) Comparative Fit Index (CFI) > 0.90, (b) Root Mean Square Error of Approximation (RMSEA) < 0.10, and (c) Standardized Root Mean Square Residual (SRMR) < 0.08, consistent with recommended thresholds (31–33). Models were considered acceptable if at least two of the three fit indices met threshold levels. In addition, we required all standardized factor loadings to be at least 0.40 to retain an item (32). Due to the large sample size, we did not use the chi-square statistic as a criterion for model fit, as even well-fitting models can produce statistically significant chi-square values in large samples. This approach aligns with Bashkov and Coggeshall’s (30) recommendation to prioritize approximate fit indices (e.g., CFI, RMSEA, SRMR) over exact fit tests in such cases.

Final models were re-estimated in the Validation Group using the same criteria. To assess local model fit, we examined correlation residuals—defined as the difference between observed and model-implied correlations. Following recommendations by Kline (34), scales were considered acceptable if no more than one pairwise residual exceeded 0.10.

Finally, in the external stage, we assessed convergent and discriminant validity by correlating the 16 subscales with each other and with established measures of related constructs, including burnout, stress, general health, well-being (WHO-5), PERMA, job satisfaction, and others. These analyses were used to evaluate whether the new scales behaved in theoretically expected ways in relation to other subscales and external variables.

## 3 Results

**Figure 1:**
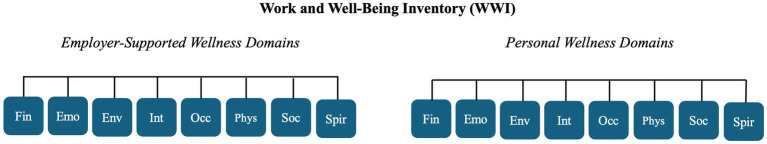
Organization of the Work and Well-being Inventory (WWI). Fin, Financial; Emo, Emotional; Env, Environmental; Int, Intellectual; Occ, Occupational; Phys, Physical; Soc, Social; Spir, Spiritual. Each dimension was modeled and validated independently within employer-supported and personal wellness domains; no higher-order latent WWI factor was specified or tested.

We first tested the factor structure of each subscale using CFAs in the Developmental Group (*n* = 474). Model fit was evaluated using three indices: CFI, RMSEA, and SRMR. Scales were considered to have adequate fit if they met at least two of the three thresholds (CFI > 0.90, RMSEA < 0.10, SRMR < 0.08). Additionally, items were required to have factor loadings ≥ 0.40 to be retained. Results are presented in Tables 2, 3, and a conceptual figure of the organizational structure is presented in Figure 1.

**Table 2:** Developmental group fit statistics.

Domains	No. of items	CFI > 0.90	RMSEA < 0.1	SRMR < 0.08	Loadings	Cronbach’s alpha
Work domains						
Financial	6 items	0.96	0.08	0.03	All above 0.4	0.83
Emotional	7 items	0.97	0.08	0.03	All above 0.4	0.88
Environmental	7 items	0.93	0.10	0.04	All above 0.4	0.82
Intellectual	7 items	0.91	0.15	0.05	All above 0.4	0.89
Occupational	7 items	0.96	0.09	0.03	All above 0.4	0.80
Physical	7 items	0.98	0.06	0.02	All above 0.4	0.89
Social	7 items	0.95	0.10	0.03	All above 0.4	0.87
Spiritual	6 items	0.95	0.11	0.04	All above 0.4	0.84
Personal domains						
Financial	7 items	0.92	0.10	0.05	All above 0.4	0.78
Emotional	7 items	0.95	0.08	0.03	All above 0.4	0.82
Environmental	7 items	0.93	0.08	0.04	All above 0.4	0.76
Intellectual	7 items	0.95	0.07	0.04	All above 0.4	0.75
Occupational	7 items	0.95	0.13	0.03	All above 0.4	0.91
Physical	6 items	0.92	0.15	0.04	All above 0.4	0.84*
Social	7 items	0.98	0.05	0.03	All above 0.4	0.80
Spiritual	4 items	0.97	0.13	0.04	All above 0.4	0.78*

* Item(s) removed from original scale due to insufficient factor loadings.

**Table 3:** Validation group fit statistics.

Domains	No. of items	CFI > 0.90	RMSEA < 0.1	SRMR < 0.08	Loadings	Cronbach’s alpha
Work domains						
Financial	6 items	0.97	0.07	0.03	All above 0.4	0.79
Emotional	7 items	0.98	0.06	0.02	All above 0.4	0.87
Environmental	7 items	0.95	0.08	0.03	All above 0.4	0.80
Intellectual	7 items	0.90	0.13	0.05	All above 0.4	0.85
Occupational	7 items	0.98	0.06	0.02	All above 0.4	0.84
Physical	7 items	0.96	0.08	0.03	All above 0.4	0.82
Social	7 items	0.93	0.10	0.04	All above 0.4	0.82
Spiritual	6 items	0.93	0.11	0.04	All above 0.4	0.83
Personal domains						
Financial	7 items	0.94	0.09	0.04	All above 0.4	0.80
Emotional	7 items	0.99	0.05	0.03	All above 0.4	0.83
Environmental	7 items	0.94	0.07	0.04	All above 0.4	0.75
Intellectual	7 items	0.94	0.07	0.04	All above 0.4	0.75
Occupational	7 items	0.93	0.13	0.04	All above 0.4	0.89
Physical	6 items	0.93	0.13	0.04	All above 0.4	0.82*
Social	7 items	0.98	0.06	0.03	All above 0.4	0.82
Spiritual	4 items	0.96	0.19	0.05	All above 0.4	0.79*

* Item(s) removed from original scale due to insufficient factor loadings.

In the Developmental Group, 14 of the 16 scales met all three fit criteria. Two scales, physical and spiritual (both in the personal domain), initially failed to meet loading criteria. One item from the physical subscale (“I avoid smoking or using tobacco products”) and two items from the spiritual subscale (“I live my life in a way that matches my values” and “I treat people who have different values with respect”) were removed due to factor loadings below 0.40. After item removal, all final models demonstrated acceptable model fit, with all item loadings ≥ 0.40. Internal consistency reliability estimates (Cronbach’s *α*) ranged from 0.75 to 0.91 across the subscales.

To assess the consistency of reliability across organizations, Cronbach’s alpha coefficients were calculated separately within each of the four participating organizations (not shown in tables). Across subscales, internal consistency estimates were highly comparable across organizations. In the employer-supported domain, alpha coefficients ranged from 0.79 to 0.90, with between-organization variability generally small (range of *α* differences ≤0.08). In the personal domain, alpha coefficients ranged from 0.72 to 0.92, again with modest variability across organizations (typically ≤0.07), with the exception of the spiritual subscale (range = 0.11). Overall, these findings suggest that the WWI subscales demonstrate stable internal consistency across organizational contexts.

We then tested the refined models in the Validation Group (*n* = 475), using the same criteria. As shown in Tables 2, 3, model fit indices remained acceptable across all 16 subscales. All items retained had loadings above 0.40, and reliability estimates ranged from 0.75 to 0.89, confirming stability of the scale structure across samples.

To further evaluate local model fit, we examined correlation residuals (i.e., the difference between observed and model-implied correlations) for each subscale in the Validation Group. Residuals are summarized in Table 4. Following Kline’s (34) guidelines, scales were considered acceptable if no more than one residual exceeded 0.10. All 16 subscales met this criterion, with either zero or one residual above 0.10, indicating minimal local misfit. Intercorrelations among the 16 subscales are presented in Table 5. As expected, scales within the same domain (employer-supported or personal) were generally more strongly correlated with one another than with scales from the other domain, supporting conceptual coherence of the domain structure. Final WWI items are provided in Appendix A.

**Table 4:** Correlation residual ranges.

Domain name	Ranges		
	Min	Max	No. > 0.1
Financial-work	−0.07	0.09	0
Emotional-work	−0.06	0.09	0
Environmental-work	−0.08	0.06	0
Intellectual-work	−0.05	0.11	1
Occupational-work	−0.10	0.18	1
Physical-work	−0.04	0.07	0
Social-work	−0.05	0.11	1
Spiritual-work	−0.07	0.09	0
Financial-personal	−0.10	0.12	1
Emotional-Personal	−0.05	0.11	1
Environmental-personal	−0.07	0.09	0
Intellectual-personal	−0.06	0.12	1
Occupational-personal	−0.06	0.09	0
Physical-personal	−0.06	0.12	1
Social-personal	−0.06	0.05	0
Spiritual-personal	−0.06	0.12	1

**Table 5:** Correlations between measures.

Domains			1	2	3	4	5	6	7	8	9	10	11	12	13	14	15	16
Work Domains	1	Financial	-															
	2	Emotional	0.697	-														
	3	Environmental	0.699	0.893	-													
	4	Intellectual	0.718	0.709	0.731	-												
	5	Occupational	0.647	0.785	0.759	0.669	-											
	6	Physical	0.728	0.697	0.720	0.698	0.690	-										
	7	Social	0.651	0.834	0.860	0.706	0.742	0.679	-									
	8	Spiritual	0.684	0.773	0.802	0.727	0.761	0.710	0.801	-								
Personal Domains	9	Financial	0.479	0.380	0.348	0.395	0.390	0.349	0.358	0.349	-							
	10	Emotional	0.490	0.560	0.564	0.538	0.603	0.503	0.537	0.558	0.555	-						
	11	Environmental	0.473	0.483	0.482	0.525	0.510	0.462	0.455	0.480	0.581	0.708	-					
	12	Intellectual	0.460	0.476	0.454	0.476	0.557	0.472	0.467	0.498	0.540	0.773	0.673	-				
	13	Occupational	0.609	0.761	0.759	0.615	0.826	0.610	0.672	0.690	0.394	0.634	0.515	0.581	-			
	14	Physical	0.278	0.296	0.284	0.274	0.322	0.304	0.241	0.270	0.500	0.538	0.626	0.514	0.360	-		
	15	Social	0.451	0.542	0.546	0.508	0.600	0.502	0.570	0.569	0.516	0.770	0.715	0.706	0.579	0.498	-	
	16	Spiritual	0.263	0.193	0.190	0.237	0.288	0.253	0.175	0.283	0.354	0.484	0.427	0.449	0.338	0.309	0.486	-

All correlations were significant at the *p* < 0.001 level.

To evaluate the external validity of the new scales, we examined correlations with established measures of well-being and related constructs (Table 6). In the employer-supported wellness domain, subscales demonstrated strong positive associations with indicators of flourishing (e.g., PERMA, WHO-5) and job satisfaction, and negative associations with stress, burnout, and intrusion. For example, the emotional (r = 0.615), environmental (r = 0.613), and occupational (r = 0.605) subscales had large positive correlations with PERMA, and moderate to large negative correlations with burnout (rs = −0.553 to −0.618).

**Table 6:** Correlations between measures and other assessments.

Domains		WHO5	PERMA	Burnout	Gen Health	Job Satis.	Control Autonomy	Stress	Recommend	Intrusion	Connection
Work domains	Financial	0.400	0.499	−0.400	−0.237	0.446	−0.523	−0.304	−0.563	−0.420	0.452
	Emotional	0.469	0.615	−0.553	−0.239	0.604	−0.701	−0.382	−0.661	−0.618	0.564
	Environmental	0.471	0.613	−0.583	−0.236	0.607	−0.656	−0.393	−0.708	−0.647	0.587
	Intellectual	0.383	0.502	−0.413	−0.260	0.425	−0.522	−0.297	−0.545	−0.472	0.451
	Occupational	0.444	0.605	−0.440	−0.232	0.542	−0.610	−0.312	−0.602	−0.434	0.509
	Physical	0.361	0.472	−0.381	−0.179	0.402	−0.511	−0.276	−0.531	−0.416	0.451
	Social	0.387	0.550	−0.434	−0.205	0.484	−0.622	−0.295	−0.602	−0.464	0.592
	Spiritual	0.379	0.528	−0.411	−0.210	0.461	−0.593	−0.275	−0.604	−0.430	0.504
Personal domains	Financial	0.416	0.351	−0.228	−0.350	0.178	−0.279	−0.287	−0.216	−0.236	0.220
	Emotional	0.592	0.503	−0.432	−0.330	0.334	−0.412	−0.439	−0.336	−0.440	0.382
	Environmental	0.511	0.409	−0.335	−0.403	0.245	−0.353	−0.358	−0.278	−0.385	0.317
	Intellectual	0.456	0.443	−0.327	−0.300	0.233	−0.341	−0.313	−0.285	−0.316	0.291
	Occupational	0.557	0.725	−0.618	−0.244	0.675	−0.591	−0.437	−0.665	−0.578	0.521
	Physical	0.524	0.308	−0.304	−0.504	0.146	−0.239	−0.384	−0.131	−0.313	0.184
	Social	0.508	0.474	−0.342	−0.311	0.285	−0.383	−0.340	−0.333	−0.362	0.429
	Spiritual	0.320	0.264	−0.213	−0.186	0.112	−0.131	−0.203	−0.137	−0.152	0.151

All correlations were significant at the *p* < 0.001 level.

In the personal wellness domain, the emotional (r = 0.592), occupational (r = 0.557), and physical (r = 0.524) subscales were most strongly associated with overall well-being and health outcomes. These patterns were consistent with theoretical expectations and support the construct validity of the employer-supported and personal wellness scales.

## 4 Discussion

Results demonstrate psychometric validation of a novel measure of employee well-being, conceptualized as eight dimensions of wellness within two contexts: a person’s overall wellness and their employer-supported wellness. The sample in this study included behavioral healthcare professionals across four states. Our results reveal that a 106-item version of the WWI showed strong structural validity, internal reliability, and convergent and discriminant validity.

Using CFA, the factor structure was examined and found acceptable for each of 16 subscales separately, such that scales for each dimension can be flexibly administered independently of the others, depending on organizational and/or research goals. This is a marked difference from existing validated wellness inventories, in which a one-factor model supports a single, holistic dimension of wellness [e.g., Wellness Inventory (11)]. This difference likely stems from the initial assumption that, although correlated, wellness in one dimension need not imply wellness in other dimensions. Additionally, the specificity of the items within each dimension ensures that results are actionable, which potentially decreases the likelihood that items across dimensions correlate. For example, whether an organization protects employees from tobacco exposure (an item within the environmental dimension of the employer-supported wellness domain) may have no bearing on whether the same organization offers ongoing educational and training opportunities (an item within the intellectual dimension of the employer-supported wellness domain).

One exception to otherwise acceptable model fit was the spiritual subscale in the personal domain within the validation sample, which demonstrated an elevated RMSEA. This subscale includes a relatively small number of items, resulting in low model degrees of freedom, a condition under which RMSEA is known to be inflated. Despite this, other fit indices (CFI and SRMR) met recommended thresholds, and all retained items demonstrated adequate factor loadings, supporting the structural validity of the scale. These findings suggest that the elevated RMSEA should be interpreted with caution. Nevertheless, future research should further evaluate this subscale, including the potential addition or refinement of items to improve model fit and stability.

Following the examples of Bashkov and Coggeshall (30), we tested convergent and discriminant validity to provide evidence of construct validity. Bivariate correlations between subscales and external measures are consistent with expectations based on current understandings of occupational wellness research.

An examination of Table 5 shows relatively higher correlations among scales within domains than between domains, which would be expected from a conceptual standpoint and speaks to the convergence of subscales and discriminant validity. Intercorrelations among employer-supported wellness subscales were all strong, ranging from 0.65 to 0.89. Among personal wellness subscales, intercorrelations were moderate to strong, ranging from 0.31 to 0.77. With the exception of correlations between employer-supported wellness subscales and the occupational wellness dimension of the personal domain (which, not surprisingly, showed strong associations), correlations between employer-supported wellness subscales and personal wellness subscales were generally low to moderate, ranging from 0.19 to 0.60. Further supporting validity, intercorrelations between subscales remain some distance from unity, suggesting they are not identical constructs, even among the highly correlated employer-supported wellness subscales.

Convergent validity is also demonstrated by correlations between WWI subscales and external measures. Table 6 shows that all 8 dimensions of wellness in the employer-supported domain are at least moderately correlated with the following work-related measures in the expected direction: PERMA (r range from 0.47 to 0.62), the Control/Autonomy composite measure (r range from 0.51 to 0.70), the Intrusion of work into private life subscale (r range from −0.41 to −0.64), and the single item measures of job satisfaction (r range from 0.40 to 0.61) and whether the respondent would recommend their job to others (r range from 0.53 to 0.71). As well, the degree to which respondents feel connected to others (assessed with a 3-item index) correlates with all employer-supported wellness dimensions in the expected direction but is particularly associated with subscales related to social wellness (r = 0.59), environmental wellness (r = 0.59), and emotional wellness (r = 0.56). This is expected, given these subscales all assess aspects of the socio-emotional climate of a workplace and further speaks to construct validity.

Discriminant validity is provided by examining correlations between subscales and measures more distally related. For example, correlations between a person’s general health and the employer-supported subscales are relatively lower (r range from 0.18 to 0.26) compared to correlations between general health and personal subscales (r range from 0.18 to 0.50), as expected given that wellness at work is one of many factors which contributes to a person’s health. Similarly, the spiritual wellness dimension within the personal domain correlates relatively weakly with all external workplace wellness measures, excluding general health (r range from 0.11 to 0.32), whereas the occupational and emotional wellness subscales in the personal domain are more highly associated with external workplace measures (occupational subscale r range = 0.44 to 0.73; emotional subscale r range = 0.33 to 0.59). This would be expected if employer-supported wellness actions contribute more to a person’s general occupational and emotional wellness than to their spiritual wellness. These demonstrations of convergent and discriminant validity show that the WWI subscales operate as expected from the existing literature, which supports the inventory’s validity.

### 4.1 Measure strengths

The WWI was developed based on existing literature from the fields of positive psychology, occupational health, and systems science. It adopts a framework based on the eight dimensions of wellness, which is both widely used across sectors and highly interpretable. The measure pays equal attention to personal wellness (that is, wellness outside of work), and workplace or employer-supported wellness as complementary factors. The entire measure takes 10 min or less to complete, and researchers or organizations may reduce that time further by selecting specific subscales based on project goals. Each subscale can be analyzed and interpreted independently of the others, supporting flexible administration. As this study shows, the WWI exhibits strong psychometric properties, with model fit statistics exceeding thresholds for adequacy. Relationships among measures are aligned with theory and expectation, demonstrating convergent and discriminant validity of the measure’s constructs.

### 4.2 Limitations

This study has several methodological limitations. Although the sample was large and represented a diverse range of professional roles (e.g., clinicians, administrators, IT professionals), a key limitation of these analyses is that the sample was drawn exclusively from behavioral health organizations. As such, the generalizability of findings may be limited to similar healthcare and public health contexts, and the psychometric properties of the WWI may differ in other occupational settings with distinct workplace structures, demands, and turnover patterns. The sample was also predominantly White, as our demographics mirror those of the regions in which the organizations operate. As with any study that requests volunteers and relies on self-report surveys, self-selection and response biases are potential limitations. We should note, however, that our response rates ranged from 41% to over 80%, depending on organization. These are relatively high rates of engagement which should limit potential self-selection bias. The cross-sectional design of our study also precluded a test of the measure’s temporal stability; future research might seek to establish test–retest reliability. It should also be noted that we did not formally test measurement invariance across organizations or occupational roles; future studies should evaluate whether the WWI demonstrates configural, metric, and scalar invariance across diverse groups. Finally, the WWI itself contains no reverse-coded items, which may increase susceptibility to acquiescence bias and contribute to common method variance. Although other included measures in the survey battery contained both positively and negatively worded items (requiring participants to remain attentive to item content) this does not fully eliminate the potential for response bias within the WWI. Future iterations of the instrument may benefit from incorporating reverse-coded or methodologically varied items to further reduce susceptibility to such biases.

### 4.3 Significance

Beyond demonstrating acceptable psychometric properties, the WWI contributes to the employee well-being literature by operationalizing wellness as a multidimensional construct that integrates both personal and employer-supported domains. This distinction is theoretically important because it recognizes employee well-being as emerging from an interaction between individual factors and organizational systems, rather than locating responsibility solely within the individual worker. The inventory therefore provides researchers with a framework for examining how specific workplace conditions and aspects of one’s personal life may differentially influence dimensions of wellness and how these dimensions, in turn, may differentially relate to outcomes such as burnout, engagement, retention, job satisfaction, and overall health. Practically, the WWI’s multidimensional structure offers organizations and practitioners actionable information that can guide workplace wellness assessment and intervention. Rather than producing only a global well-being score, the inventory identifies specific personal and employer-supported domains in which employees may be thriving or struggling, thereby allowing leaders to tailor interventions to targeted areas such as occupational, emotional, social, or environmental wellness. Accordingly, the WWI may support organizational decision-making, program development, and evaluation of wellness initiatives over time. Analyzing pre/post changes across relevant subscales would effectively measure the impact of a specific wellness initiative undertaken by an organization. In this way, the WWI will help organizations move beyond generalized wellness programming toward more precise, data-informed approaches to improving employee well-being and workplace culture.

### 4.4 Future directions

This novel assessment has several potential applications. First, baseline scores could provide a roadmap for leaders, administrators, and wellness champions from organizations seeking to improve employee retention and/or advance a culture of wellness. Second, the WWI provides researchers with a validated instrument to expand scientific knowledge of how specific aspects of the workplace, organized across the eight dimensions of wellness, impact human health and wellness. Future research should explore how the WWI relates to health and wellness measures across different professional settings beyond behavioral healthcare. Studies using the WWI should also investigate individual differences in how employees respond to workplace wellness initiatives, and whether certain dimensions are more or less important within particular demographic groups (e.g., senior vs. junior; men vs. women). As well, studies should evaluate the impact of common employee wellness initiatives on the WWI’s wellness dimensions, including whether initiatives improve only employer-supported wellness dimensions or whether they impact personal wellness overall.

## 5 Conclusion

This study represents the first psychometric evaluation of the WWI, a measure of personal and employer-supported well-being. To our knowledge, the WWI is the only validated measure of employee well-being that adopts an ‘eight dimension’ model of wellness. Factor structure and model fit statistics support flexible administration and parsimonious interpretation of eight subscales directly corresponding to eight dimensions of wellness. The instrument is designed for both researchers and organizations seeking to broaden their understanding of holistic wellness and evaluating initiatives designed to improve workplace culture. Future research examining impacts of workplace wellness initiatives on WWI subscales, and studies to identify individual differences in responses to those initiatives would further strengthen the WWI’s application.

## Data Availability

The data analyzed in this study is subject to the following licenses/restrictions: no restrictions. Requests to access these datasets should be directed to Christine E. Garver-Apgar, Christine.Garver-Apgar@cuanschutz.edu.
